# Primary Ovarian Non-Hodgkin’s Lymphoma: An 18-year Retrospective Institutional Study and Review of Literature from India

**DOI:** 10.7759/cureus.40685

**Published:** 2023-06-20

**Authors:** Tara Rajendran, Jyoti R Kini, Krishna Prasad

**Affiliations:** 1 Department of General Medicine, Kasturba Medical College-Mangalore, Manipal Academy of Higher Education (MAHE-Manipal), Mangalore, IND; 2 Department of Music, Faculty of Fine Arts, Annamalai University, Chidambaram, IND; 3 Department of Pathology, Kasturba Medical College-Mangalore, Manipal Academy of Higher Education (MAHE-Manipal), Mangalore, IND; 4 Department of Medical Oncology, Mangalore Institute of Oncology, Mangalore, IND

**Keywords:** india, primary extra-nodal non-hodgkin’s lymphoma, ovary, lymphoma, non hodgkin lymphoma

## Abstract

Background: Primary lymphomas of the female reproductive tract are rare and the ovarian extranodal presentation of non-Hodgkin's lymphoma (NHL) accounts for only 0.5% of all NHLs and 1.5% of all ovarian malignancies.

Methods: We retrospectively reviewed the institutional medical oncology database for newly diagnosed NHL cases between 1999 and 2017. We aimed to study the clinical characteristics, pathology, and outcome of primary ovarian non-Hodgkin’s lymphoma (NHL) cases presented to our institution.

Results: We identified three patients (3.7% of extranodal NHLs and 0.85% of all NHL patients) with primary ovarian NHL from 350 NHL patient records. They underwent total abdominal hysterectomy and bilateral salpingo-oophorectomy followed by six to eight cycles of (rituximab, adriamycin, cyclophosphamide, vincristine, prednisolone (R-CHOP/CHOP), and they attained complete remission.

Conclusion: Given the heterogeneity of cancer incidence in India and the absence of state-wise cancer registries, our study argues a pressing need to develop a national representative registry for NHL for accurate incidence, mortality, and survival data. Additionally, fertility preservation is an important issue that must be discussed with women of fertile age and the parents of children.

## Introduction

Non-Hodgkin's lymphoma (NHL) is a heterogeneous collection of malignancies emerging from lymphoid tissues, with over 80 subtypes [1], and it accounts for 3% of cancer cases worldwide [2]. 2020's Globocan report estimates NHL's age-standardized mortality rate to be 2.6 per 100,000 worldwide. According to the National Cancer Registry Program (NCRP), the Indian Council of Medical Research (ICMR) reports that the estimated incidence rate of NHL in India is 2-3 per 100,000 people. For the year 2020, ICMR approximate NHL (38,098, 28.7%) to contribute a maximum to lymphoid and hematopoietic system-related cancers [3]. The crude incidence rate generated by the five urban and one rural population-based-cancer registry finds that NHL is emerging as a notable cause of cancer mortality in India, increasing the national cancer burden [4].

Primary lymphomas of the female reproductive tract are exceedingly unusual, and the ovarian extranodal presentation of NHL accounts for only 0.5% of all NHLs and 1.5% of all ovarian malignancies [5]. However, ovaries are the most common [6] anatomic location in the gynecologic tract. Initially, the origin of malignant ovarian lymphoma was perplexing as it was believed that no normal lymphoid tissue was present in the ovary [7]. In 1963, a study of 35 ovarian lymphoma cases from the ovarian tumor registry of the American Gynecological Society discovered that lymphoid aggregates are present in the hilus and medulla in normal ovaries [8]. A clinicopathologic analysis of 39 cases of malignant ovarian lymphomas conducted in 1993 found benign lymphoid aggregates in roughly 50% of the normal ovaries [9].

There is heterogeneity in state-wise cancer incidence within India, and there are gaps in incidence and mortality registration, making it incomplete and inaccurate [10]. This article aims to report our experience managing this rare gynecologic malignancy and review other published primary ovarian NHL articles from India that describe the histopathology and/or clinical characteristics. 

## Materials and methods

We searched for newly diagnosed primary ovarian NHL cases established by histopathology between 1999 and 2017, identified from the medical records department and medical oncology electronic database at Kasturba Medical College and Hospital, Mangalore, India. We excluded the patients who discontinued the treatment. The Institutional Ethics Committee of Kasturba Medical College, Mangalore, Manipal University issued approval IEC KMC MLR 05-16/120.

Patients had a detailed physical examination, complete blood and biochemistry panels, renal function tests (RFT), chest radiographs, and computed tomography (CT) of the abdomen and pelvis. Additional studies including fluorodeoxyglucose-positron emission tomography were conducted as clinically indicated. We followed the World Health Organization (WHO) guidelines for histologic classification and the National Comprehensive Cancer Network - International Prognostic Index for Prognosis (NCCN-IPI). 

Disease progression is defined as the enlargement of an existing site or disease development in a previously uninvolved site. As per the revised response criteria for malignant lymphomas (2016), complete remission is defined as the disappearance of all evidence of disease. The overall survival (OS) is defined as the length of time from the initial diagnosis date that patients diagnosed with the disease are still alive. Failure-free survival is defined as the absence of relapse, non-relapse mortality, or the addition of another anti-neoplastic systemic therapy from the initiation of treatment. 

## Results

In the institutional review, we retrieved 350 newly diagnosed NHL patient records between 1999 and 2017. A total of 80 patients (22.8%) were identified with primary extra-nodal involvement. Three patients (3.7%) had primary ovarian involvement, which was 0.85% of all NHL patients. Pertinent patient characteristics are demonstrated in Table 1. The median age is 50 (32-59). One patient was pre-menopausal, and two of them were post-menopausal. All patients presented with pelvic complaints, and none were incidental findings. Two patients presented with abdominal pain and one with a pelvic mass. One patient was human immunodeficiency virus (HIV)-infected, and she reported generalized weakness alongside pelvic complaints (body mass index: 16.6). Only one patient had B symptoms (fever, night sweats, and weight loss without dieting) (Table 1).

**Table 1:** Institutional review of clinical characteristics, response to treatment, and outcome. IHC, Immunohistochemistry; AAS, Ann Arbor Stage; NCCN-IPI, The National Comprehensive Cancer Network-International Prognostic Index; OS(Mo), Overall Survival in months; FFS (Mo), Failure free survival in months; DLBCL, Diffuse Large B-cell Lymphoma; NA, Data Not Available in the records; TAHBSO, Total abdominal Hysterectomy Bilateral Salpingo-Oophorectomy, RCHOP, Rituximab, Adriamycin Cyclophosphamide Vincristine Prednisolone; NED, No evidence of disease; DLCL, Diffuse Large Cell Lymphoma; HIV, Human Immunodeficiency Virus.

Sl. No	Age	Menstrual status	Significant Medical History	Symptoms	Side	Size (Centimeters)	Pathology	IHC	LDH	CA-125 (in milligram)	Stage AASS	NCCN-IPI	Surgical treatment	1st therapy	Response	Follow-up	OS (Mo)	FFS (Mo)
1	58	Post- menopausal	Completed Tuberculosis treatment	Abdominal Pain	Left	22x22x10	DLBCL	CD3+, CD20+, LCA+, CK-	NA	NA	IV AE	2	TAH, BSO	6 RCHOP	CR	Alive NED	147	147
2	59	Post-menopausal	None	Left Ileac fossa pain for a week	Left	8x6x4.5	DLBCL	CD20+, CD 45+	539	9.29	I AE	1	TAH, BSO	6 RCHOP	CR	Alive NED	49	49
3	32	Pre-menopausal	HIV	Mass per abdomen Generalized Weakness	Right	20x4x2	DLBCL-Germinal Centre type	CD10+ CD20+ BCL6+, CD3-, BCL12-, Ki 67:80%	NA	NA	1BE	0	TAH, BSO	8CHOP	CR	Alive NED	4	4

All three patients were diagnosed with ovarian lymphoma after the total abdominal hysterectomy and bilateral salpingo-oophorectomy. All three patients had unilateral ovarian involvement. Two cases involved the left ovary, and one case involved the right ovary. Ovarian masses were grossly identified, and they measured 22x22x10, 8x6x4.5, and 20x4x2 centimeters. One patient (stage 4) had both ovary and liver involvement, and the rest (two patients) had exclusive ovarian involvement. There was no lymph node involvement in all three patients.

**Figure 1:**
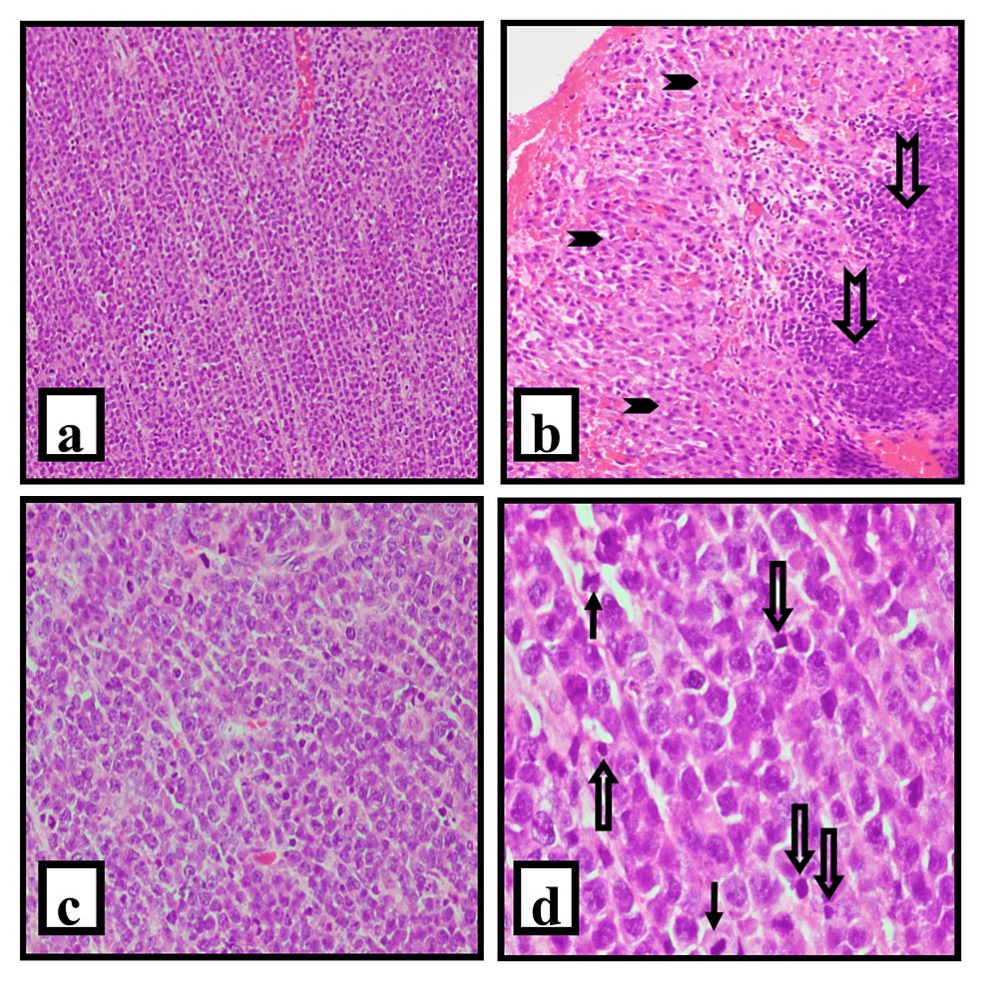
Microphotograph of primary ovarian diffuse large B-cell lymphoma; hematoxylin and eosin (H&E) stain. (a): Section shows replacement of ovarian parenchyma by a diffuse infiltration of monotonous population of lymphoid cells (H&E, 10x). (b): Atypical lymphoid infiltrate sparring the corpus luteum (H&E, 10x). The solid arrowhead shows the corpus luteum and the notched arrow shows the atypical lymphoid infiltrate. (c) Medium to large lymphoid cells with oval to round vesicular nuclei, some with conspicuous nucleoli, and scant to moderate pale eosinophilic cytoplasm (H&E, 20x). (d) Cords of malignant lymphoid cells with irregular nuclear membrane, interspersed scattered mitotic figures, and apoptotic bodies (H&E, 40x). The arrow shows mitosis and the hollow arrow shows apoptotic bodies Image Credit: Jyoti R Kini

Microscopic examination revealed ovarian parenchyma infiltrated with a tumor composed of a monomorphous population of medium to large lymphoid cells with high nucleo-cytoplasmic ratio, oval to round vesicular nuclei with irregular nuclear margins, and some with prominent nucleoli and a scant to moderate amount of pale eosinophilic cytoplasm. Atypical mitotic figures were seen. These tumor cells were seen predominantly in a diffuse pattern (Figure 1). However, the neoplastic cells were arranged in cords, trabeculae, and nests at places. One of the cases showed sclerotic stroma. Reticulin stain showed fine reticulin fibers surrounding individual and groups of tumor cells. In patient 3, we found focal epithelioid cell granulomas along with the tumor. Large areas of necrosis were seen in one of the cases (Figure 1).

**Figure 2:**
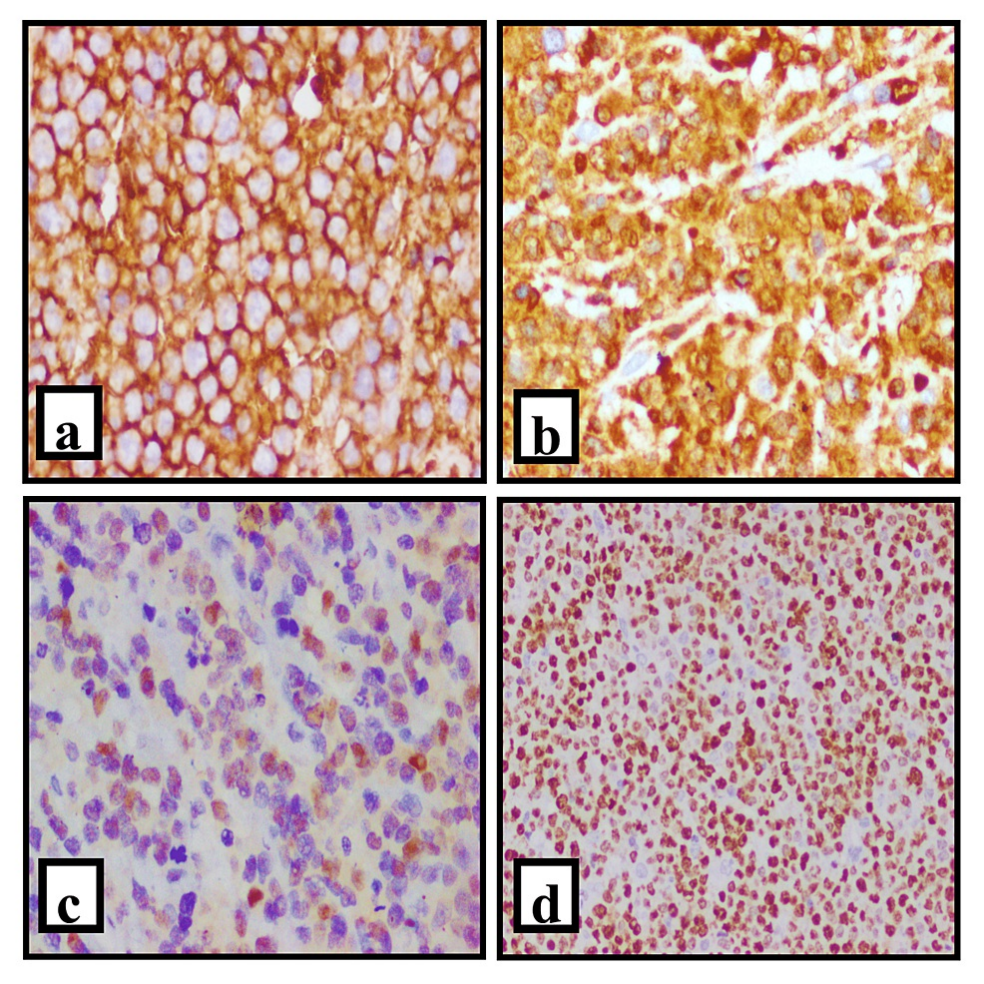
Microphotograph of primary ovarian NHL-DLBCL, germinal center type, immunohistochemistry. (a): Strong membranous positivity of the lymphoid cells with CD 45(LCA) (40x). (b): Membranous positive staining with CD 20 (40x). (c): Positive nuclear staining with BCL6. (d): Approximately 80% ki 67 proliferation index (40x). DLBCL, Diffuse Large B cell Lymphoma Image Credit: Jyoti R Kini

An immunohistochemistry panel was done in all patients. All three cases were positive for CD 20 (Figure 2). Diffuse large B-cell lymphoma is the histological type of all three patients. Patients were staged according to the Ann Arbor staging system. We had patients with low risk to low intermediate risk according to NCCN-IPI (Figure 2).

Medical oncologist planned the management after discussing with the hemato-pathologist, radiologist, gynecologist, and surgical oncologist. Patients were treated with six cycles of RCHOP or CHOP (rituximab, adriamycin, cyclophosphamide, vincristine, and prednisolone). We evaluated the response using positron emission tomography or computed tomography, depending on the patient’s financial affordability. One of the patients could not afford rituximab due to her financial constraints. Clinical follow-up spanned from 4 to 147 months (Mean: 66.6 months). All three patients achieved complete remission. At the last follow-up, all patients were alive, relapse, and disease free.

## Discussion

In India, the most common location for extra-nodal involvement of NHL is the tonsil [11,12]. The female reproductive tract is comparatively an uncommon extra-nodal site for NHL, but within the gynecological tract, the ovary is one of the most common locations [6]. Ovarian NHL usually replaces the entire normal parenchyma but tends to spare the evolving follicles, corpora lutea, and albicans, as shown in our case (Figure 1b). Diffuse large B cell lymphoma is the most common histological category, as seen in our series and in the literature. Burkitt lymphoma involving the ovary is frequently found in children and young adults, especially where it is endemic and is most often bilateral. Follicular lymphoma is usually seen in older patients. Other rarer lymphomas include anaplastic large cell lymphoma and B and T lymphoblastic lymphomas. Histological mimics include granulosa cell tumors, carcinoma, and spindle cell sarcoma, as the neoplastic lymphoid cells may be pleomorphic or elongated and arranged in cords/ nests or a storiform pattern. Immunohistochemistry and molecular techniques are thus a part of the diagnostic armamentarium [13].

We studied similar retrospective institutional reviews/case series published in India; the literature search yielded 14 publications that discussed 20 cases. The summary is listed in Table 2. 

**Table 2:** Clinicopathologic characteristics of the published cases of primary ovarian NHL between 1990 and 2020. IPI, International Prognostic Index; OS, Overall Survival; TAH, Total Abdominal Hysterectomy; BSO, Bilateral Salpingo-Oophorectomy; COP, Cyclo phosphamide Vincristine, Prednisolone; CR, Complete Remission; RCHOP, Rituximab, Adriamycin Cyclophosphamide Vincristine Prednisolone; CHOP, Adriamycin Cyclophosphamide Vincristine Prednisolone; R, Relapse; RT, local radiotherapy; DSBCL, Diffuse Small B cell Lymphoma; DLBCL, Diffuse Large B cell Lymphoma; PR, Partial response; MTP, Medical Termination of Pregnancy; LN, Lymph Node; LBL, Lymphoblastic Lymphoma; DLCL, Diffuse Large cell Lymphoma; MCP 842, Multi-Center Protocol - 842 Protocol; BFM 90, Berlin-Frankfurt-Munster 90 protocol; GCB, Germinal Centre B cell-like. *Ann Arbor Stage - Data not available

REFERENCE	AGE	OTHER FOCUS	CLINICAL PRESENTATION	IPI	STAGE	HISTOLOGY	SURGERY	CHEMOTHERAPY	OS (months)
Advani et al. 1990 [12]	62	Bone Thyroid	-	-	-	Diffuse histiocytic	-	CHOP	PR (6) Died
	40	Base Tongue (R)	-	-	-	Diffuse undifferentiated	Surgery	COP	CR (37) RT (88)
Chawla et al. 1990 [14]	38	Appendix	Abdominal pain	-	-	Poorly differentiated Lymphocytic lymphoma	TAH, BSO	COP 6	CR
Ambulkar et al. 2003 [15]	40	-	Large pelvic mass	1	2C(FIGO)	DSBCL	Ovarian mass removed	6 CHOP	CR (24)
	24	-	Abdominal pain	1	IIIA (FIGO)	High-Grade B cell	Oophorectomy	6 CHOP	CR (6)
Lanjewar et al. 2006 [16]	28	-	Fever, Weightloss, Abdominal pain and distension, HIV	-	-	DLBCL	Left SO	Couldn’t afford	Died seven months after the diagnosis
Bangera et al. 2009 [17]	40	-	Abdominal pain, Vomiting, Diarrhea, HIV	-	-	DLCL	TAH, BSO	Refused Treatment	Died three months post-surgery
Ray et al. 2008 [18]	8	-	Pain, Abdominal mass	-	-	-	TAH, BSO	-	-
Yadav et al. 2012 [19]	28	-	Incidental, MTP	-	-	Precursor B-LBL	TAH, BSO Omentectomy, paraaortic LN dissection	Refused Treatment	Died
Kumar et al. 2012 [20]	45	-	Pain Abdomen, heaviness in the abdomen	-	IE^*^	DLBCL	TAH, BSO	6 RCHOP	CR
Khan et al. 2013 [21]	6	Endometrium	Pain Abdomen, Lump in the abdomen	-	Murphy stage 2	Burkitt	BSO, Hysterectomy	MCP 842 Protocol	-
Pandey et al. 2015 [22]	75	-	Acute left iliac fossa pain Localized guarding & peritonitis	-	-	High-grade NHL	TAH, BSO	-	-
Babu et al. 2016 [23]	41	-	Pain abdomen	2	IV BE^*^	DLBCL	TAH, BSO	6 RCHOP	CR (20)
	43	-	Abdominal swelling	1	I BE^*^	DLBCL	TAH, BSO	6 CHOP	CR (22)
	59	-	Pelvic mass	0	I AE^*^	DLBCL	TAH, BSO	6 CHOP	CR (41)
	53	-	Abdominal swelling, Pain, Abdomen	2	IV BE^*^	DLBCL	TAH, BSO	6 CHOP	CR (6)
	52	-	Pain Abdomen	2	IV AE^*^	DLBCL	TAH, BSO	6 CHOP	R (26)
Narayanan et al. 2016 [24]	15	CNS	Pain Abdomen	-	-	Pre B Lymphoblastic Lymphoma	No surgery	BFM 90	CR >10Yrs RT
Bhartiya et al. 2016 [25]	52	-	Pain, distention of Abdomen, Back pain, Generalised weakness	-	-	DLBCL	-	-	-
Zaidi et al. 2019 [26]	21	-	Pain Abdomen, Distention, Constipation	-	-	DLBCL, GCB	BSO	-	-

One of India's earliest published institutional reviews of NHL (1965) was by Desai et al. from a quaternary cancer center (Tata Memorial Hospital, Bombay) between 1941 and 1960. [11]. Three decades after this study, from the same regional cancer center, Advani et al. [12] (1990, study period 1981-1985) presented the most extensive institutional review of that time, which reported 307 (22.4%) extra-nodal NHL cases. Although they reported 32 extra-nodal cases located in the ovary and vagina combined, the clinical presentation of ovarian NHL was not mentioned separately. However, out of these 32 cases, two of the ovarian lymphomas had multiple focuses, and only those two cases had the management and survival details explained. They report diffuse poorly differentiated lymphocytic lymphoma as the most common histological type (8, 25%) among the gynecologic tract NHLs. 

Chawla et al. 1990 [14] detailed a multifocal diffuse poorly differentiated lymphocytic lymphoma patient with a bilateral ovarian mass of 20x15 centimeters each and an appendicular mass of 8x6 centimeters. The patient underwent total abdominal hysterectomy-bilateral salpingo-oophorectomy, infracolic omentectomy, and appendectomy, followed by six cycles of cyclophosphamide, vincristine, and prednisolone. The patient attained complete remission and was disease free on subsequent follow-ups. However, the number of failure-free survival months is not available. 

In 2003, Ambulkar et al. [15] reported that patients with primary ovarian NHL appear to have an identical outcome compared to any other NHL. In 2006, Lanjewar et al. [16] published the case report of the first HIV-associated primary ovarian NHL from India, providing insights into the cost of cancer care and its affordability among Indian patients. They document that several patients from low- and middle-income countries (LMICs) could not afford standard NHL combination chemotherapy and highly active antiretroviral therapy (HAART). Bangera et al. [17] present a similar case where the patient underwent surgical de-bulking but refused chemotherapy. Both these patients with HIV-associated NHL succumbed to death within a year of diagnosis. Ray et al. 2008 [18] reported an eight-year-old with a space-occupying bilateral ovarian mass who underwent total abdominal hysterectomy-bilateral salpingo-oophorectomy followed by chemotherapy; survival outcomes are not available. 

Yadav et al. [19] 2012 presented a patient with primary bilateral ovarian precursor-B lymphoblastic lymphoma, a rare histologic subtype. The patient refused to be treated, and three months later, she was diagnosed with leukemia and succumbed to the disease. Khan et al. [21] reported a 6-year-old patient, the youngest ovarian NHL patient from India, with both endometrial and bilateral ovarian involvement. She underwent bilateral salpingo-oophorectomy and hysterectomy followed by multi-center protocol (MCP) 842, cyclophosphamide, ifosfamide, doxorubicin, etoposide, ara-C, methotrexate, and vincristine with prophylactic intrathecal Methotrexate and ara-C. Jacob et al. describe the first enteropathy-associated T cell lymphoma presenting as bilateral ovarian masses [27,28]. Narayanan et al. [24] reported a post-menarchial adolescent with primary ovarian pre-lymphoblastic lymphoma with central nervous system involvement managed using high-risk BFM 90 - (Berlin-Frankfurt-Munster) protocol and radiation. The patient is reported to be in complete remission at 124 months, and this case has one of the longest-documented survivals in ovarian pre-B lymphoblastic lymphoma. In 2000, Singh et al. reported NHL with extensive involvement, including bilateral kidneys, lungs, uterus, ovaries, muscles, and bone [29]. 

According to the National Cancer Registry Program, the Indian Council of Medical Research reports that the estimated age-adjusted incidence rate of NHL in Indian women is 1.5 per 100,000. Projected trends of NHL burden in India show a steady progression from 2012 through 2035 [4]. Affordability of the current standard of NHL management is challenging for many patients in India; in our hospital, we had one patient who could not afford rituximab based regimen, and in our literature review, there were two patients with HIV-associated primary ovarian NHL, both succumbed to the disease without treatment [16,17]. In April 2004, the government of India, under the National AIDS Control Program (NACO), initiated free anti-retroviral drugs [20] for all HIV/acquired immunodeficiency syndrome (AIDS) patients. It was initially launched at eight [16] government hospitals that subsequently increased to 425 in a decade serving 0.7 million patients. In our study, we treated one HIV-associated primary ovarian NHL patient who responded well to total abdominal hysterectomy bilateral salpingo-oophorectomy with eight cycles of RCHOP and attained complete remission. 

Three primary ovarian NHL cases of pediatric/young adolescents were studied [18,21,24]. Two of them underwent total abdominal hysterectomy bilateral salpingo-oophorectomy at age six [21] and eight [18], respectively; the details on fertility preservation are not described. Narayanan et al. [24] present a ten-year failure-free survival of a 15-year-old girl with primary ovarian NHL and central nervous system involvement, managed with induction and maintenance chemotherapy and cranial irradiation, without total abdominal hysterectomy bilateral salpingo-oophorectomy. The patient restarted her regular menstrual cycles. The authors state that it is vital to make a pre-operative diagnosis as NHL responds well to chemotherapy and has a good prognosis. The field of fertility preservation is rapidly advancing, and it is an important matter to discuss with women of fertile age and parents of pre-menarchial children [30]. 

The limitation of our study is that it is a single-center, retrospective, and record-based study based on medical oncology database. We treated only three patients with primary ovarian NHL; the follow-up period was short. The results can not be generalized. 

## Conclusions

There is heterogeneity in cancer incidence, survival, and mortality within India. In addition to the absence of high-quality data on cancer incidence and mortality, lack of accessibility, health insurance, and affordability are significant challenges in cancer care. Our study argues the pressing need to develop a national representative registry of NHL at cancer centers for accurate incidence, mortality, and survival data. We recommend medical/surgical oncologists initiate a discussion on the available options for fertility preservation to eligible patients.
